# Prognostic value of ^18^F-FDG PET/CT in patients with advanced or metastatic non-small-cell lung cancer treated with immune checkpoint inhibitors: A systematic review and meta-analysis

**DOI:** 10.3389/fimmu.2022.1014063

**Published:** 2022-11-17

**Authors:** Tao Ling, Lianghui Zhang, Rui Peng, Chao Yue, Lingli Huang

**Affiliations:** ^1^ Department of Pharmacy, Suqian First Hospital, Suqian, China; ^2^ Department of Oncology, Changzhou Traditional Chinese Medicine Hospital, Changzhou, China; ^3^ Department of General Surgery, Jiangsu Cancer Hospital, Jiangsu Institute of Cancer Research, The Affiliated Cancer Hospital of Nanjing Medical University, Nanjing, China; ^4^ Department of Pharmacy, Jiangsu Cancer Hospital, Jiangsu Institute of Cancer Research, The Affiliated Cancer Hospital of Nanjing Medical University, Nanjing, China

**Keywords:** immune checkpoint inhibitor, non-small-cell lung cancer, prognosis, ^18^F-FDG PET/CT, metabolic tumor volume

## Abstract

**Purpose:**

This study aimed to investigate the value of ^18^F-fluorodeoxyglucose positron emission tomography/computed tomography (^18^F-FDG PET/CT) in predicting early immunotherapy response of immune checkpoint inhibitors (ICIs) in patients with advanced or metastatic non-small-cell lung cancer (NSCLC).

**Methods:**

A comprehensive search of PubMed, Web of science, Embase and the Cochrane library was performed to examine the prognostic value of ^18^F-FDG PET/CT in predicting early immunotherapy response of ICIs in patients with NSCLC. The main outcomes for evaluation were overall survival (OS) and progression-free survival (PFS). Detailed data from each study were extracted and analyzed using STATA 14.0 software.

**Results:**

13 eligible articles were included in this systematic review. Compared to baseline ^18^F-FDG PET/CT imaging, the pooled hazard ratios (HR) of maximum and mean standardized uptake values SUV_max_, SUV_mean_, MTV and TLG for OS were 0.88 (95% CI: 0.69-1.12), 0.79 (95% CI: 0.50-1.27), 2.10 (95% CI: 1.57-2.82) and 1.58 (95% CI: 1.03-2.44), respectively. The pooled HR of SUV_max_, SUV_mean_, MTV and TLG for PFS were 1.06 (95% CI: 0.68–1.65), 0.66 (95% CI: 0.48-0.90), 1.50 (95% CI: 1.26-1.79), 1.27 (95% CI: 0.92-1.77), respectively. Subgroup analysis showed that high MTV group had shorter OS than low MTV group in both first line group (HR: 1.97, 95% CI: 1.39-2.79) and undefined line group (HR: 2.11, 95% CI: 1.61-2.77). High MTV group also showed a shorter PFS in first line group (HR: 1.85, 95% CI: 1.28-2.68), and low TLG group had a longer OS in undefined group (HR: 1.37, 95% CI: 1.00-1.86). No significant differences were in other subgroup analysis.

**Conclusion:**

Baseline MTV and TLG may have predictive value and should be prospectively studied in clinical trials. Baseline SUV_max_ and SUV_mean_ may not be appropriate prognostic markers in advanced or metastatic NSCLC patients treated with ICIs.

**Systematic Review Registration:**

https://www.crd.york.ac.uk/prospero/display_record.php?RecordID=323906, identifier CRD42022323906.

## Introduction

Lung cancer is the second most common malignant tumor with the highest mortality rate worldwide, of which non-small-cell lung cancer (NSCLC) accounts for about 85% (1, 2). Due to the relatively insidious symptoms of early NSCLC, most diagnosis presents local spread or distant metastasis, resulting in poor prognosis. As one of the most important breakthroughs in oncology in recent years, immune checkpoint inhibitors (ICIs), characterized by programmed death 1 (PD-1), programmed death ligand 1 (PD-L1) and cytotoxic T lymphocyte associated antigen 4 inhibitors, have dramatically changed the treatment landscape, and even advanced into the first-line treatment of advanced or metastatic NSCLC (3–5). Immunotherapy can significantly improve the patient’s clinical outcome and for the first time offer hope of long-term survival for NSCLC patients. However, not all patients can benefit from immunotherapy, because only a small percentage of NSCLC patients are sensitive to ICIs (6, 7). The response rate for unscreened NSCLC patients treated with ICIs alone is usually less than 20% (3). On the other hand, unlike most traditional treatments, immunotherapy usually shows delayed response, pseudoprogression, hyperprogression and other phenomena, as well as immune related adverse events (8, 9), which brings difficulties and challenges to clinical treatment decision-making.

The complexity of immunotherapy response challenges the monitor of treatment assessment using conventional methods. Predictive biomarkers are required to determine immunotherapy achievement and survival benefit. PD-L1 expression is a typical and predictive biomarker for ICIs treatment, and patients who benefit most from immunotherapy can be selected (10). However, predictions based on PD-L1 levels are sometimes biased. Some patients without PD-L1 expression can achieve certain remission and control of lesions after immunotherapy, while some patients with PD-L1 expression cannot benefit from immunotherapy (11, 12). Tumor mutation burden is another new potential biomarker that can predict progression-free survival (PFS), but its prediction of overall survival (OS) is not reliable enough (13, 14). As the most important weapon of anti-tumor immunotherapy, tumor-infiltrating lymphocytes (TILs) CD3^+^, CD8^+^ and forkhead box protein 3 (FOXP3^+^) may be independent predictors of clinical benefit of ICIs (15–17). The most significant limitation, however, is the inability to predict efficacy from their density and location prior to treatment. Therefore, it is the focus and difficulty of ICIs treatment to find effective prognostic markers to predict immunotherapy response and screen out patients who really benefit.

With the progress of technical approaches and the wide application of ICIs, studies on dynamic monitor of treatment response and prognosis based on tissue specimen, liquid biopsy and imaging are gradually increasing. ^18^F-fluorodeoxyglucose positron emission tomography/computed tomography (^18^F-FDG PET/CT) is a functional noninvasive imaging modality based on glucose metabolism, which is of great value in cancer diagnosis, staging, efficacy judgment and prognosis evaluation (18). Malignant tumor cells proliferate and divide in an abnormally active manner, requiring more energy and leading to enhanced glycolysis. The high expression of glucose transporter type 1 can transport a large amount of glucose to meet its demand for rapid reproduction, resulting in the gradual aggregation of ^18^F-FDG in tumor cells and abnormal radioactive hyperconcentration of lesions (19). ^18^F-FDG PET/CT can reflect the systemic metabolism of malignant tumors at the molecular level (20, 21), so it can diagnose and evaluate the therapeutic efficacy of malignant tumors earlier and more accurately, and monitor survival prognosis. Several studies have shown that PET/CT metabolic parameters including the maximum standardized uptake values (SUV_max_), the mean standardized uptake values (SUV_mean_), metabolic tumor volume (MTV) and total lesion glycolysis (TLG) have important predictive value in the prognostic evaluation of malignant tumors. Some studies have found that SUV_max_ can be used as a potential predictor of ICIs response (22, 23), while other studies have not found their relationship (24). MTV is usually considered as a typical prognostic parameter that reflects tumor volume with metabolic activity and the metabolic volume of anatomical lesions. TLG includes tumor metabolic activity and tumor metabolic volume, which can fully reflect the overall metabolic characteristics of tumor lesions. Studies have proved that baseline MTV and TLG can better predict prognosis than SUV_max_ for NSCLC patients undergoing surgery, radiotherapy, chemotherapy and targeted therapy (25–28). In terms of immunotherapy, the prognostic and predictive value of these metabolic parameters in NSCLC patients treated with ICIs is still unknown. This study aimed to investigate the prognostic value of baseline ^18^F-FDG PET/CT metabolic parameters MTV, SUV_max_, SUV_mean_, and TLG for survival in advanced or metastatic NSCLC patients treated with ICIs.

## Methods

### Data source and search strategy

A comprehensive literature search for examining the value of ^18^F-FDG PET/CT in assessing immunotherapy response of patients with NSCLC was conducted in PubMed, Embase, the Cochrane Library and Web of Science databases. The keywords (MeSH, Emtree) include “positron emission tomography computed tomography”, “carcinoma, non-small-cell lung” and “immune checkpoint inhibitors”. The search strategy was combined with keywords and free words. The search was carried out without any language restriction from the inception of the databases to March 28, 2022. The bibliographies of the pertinent articles were searched to identify additional relevant studies. Full-text articles were further checked if abstracts did not provide sufficient information. Furthermore, the reference lists of related articles were scrutinized for additional studies. Reviews, case reports, letters to the editor, editorials comments, and conference abstracts were excluded.

### Study selection

The inclusion criteria were as follows: 1) patient diagnosed as stage III or IV NSCLC patients; 2) patients treated with immune checkpoint inhibitors; 3) ^18^F-FDG PET or PET/CT performed before ICIs treatment as baseline; 4) reporting metabolic parameters, such as baseline SUV_max_, SUV_mean_, MTV or TLG; 5) reporting survival data that HR and 95%CI can be directly obtained or calculated. Review articles, case reports, letters and studies with missed data or without follow-up were excluded.

### Data extraction and collection

Data were extracted from searched publications by two authors of the manuscript independently (Tao Ling and Lianghui Zhang), and then the data were collected to a pre-designed data extraction form within Microsoft Excel version 2021 (Microsoft Corporation, Seattle, Washington, USA). Any difference in retrieval and filter was settled by consensus. Detailed data were extracted from each eligible study, including the first author, year of publication, country, study designing, number of patients, TNM staging, treatment protocol, and study endpoints.

### Quality assessment

The risk of bias of the included studies was evaluated using the Newcastle-Ottawa Scale (NOS) based on three broad domains: selection of the study groups, comparability among different groups and ascertainment of either the exposure or outcome of interest (29). The total score of NOS uses a star system (maximum of nine stars) based on its assessment items. Two authors (Tao Ling and Lingli Huang) assessed the quality items and differences independently.

### Statistical analyses

The endpoints were OS and PFS. The relationship between SUV_max_, SUV_mean_, MTV, TLG and OS and PFS was evaluated in terms of the hazard ratio (HR) effect size. Univariate or multivariate HR estimates with 95% confidence intervals (CIs) were extracted directly from each study, if possible. For patients with high SUV_max_, SUV_mean_, MTV or TLG, HR greater than 1 implied worse survival, whereas HR smaller than 1 implied a survival benefit. Heterogeneity was assessed by Cochran’s Q value (and its associated p value) as well as the inconsistency index (I^2^ index). I^2^ levels of 50% or less correspond to a low heterogeneity and I^2^ levels of 50% more correspond to a high heterogeneity. Begg’s test was used to assess publication bias. *P* < 0.05 was considered statistically significant. Data from each study were analyzed using STATA 14.0 software.

## Results

### Characteristics of the eligible studies

**Figure 1:**
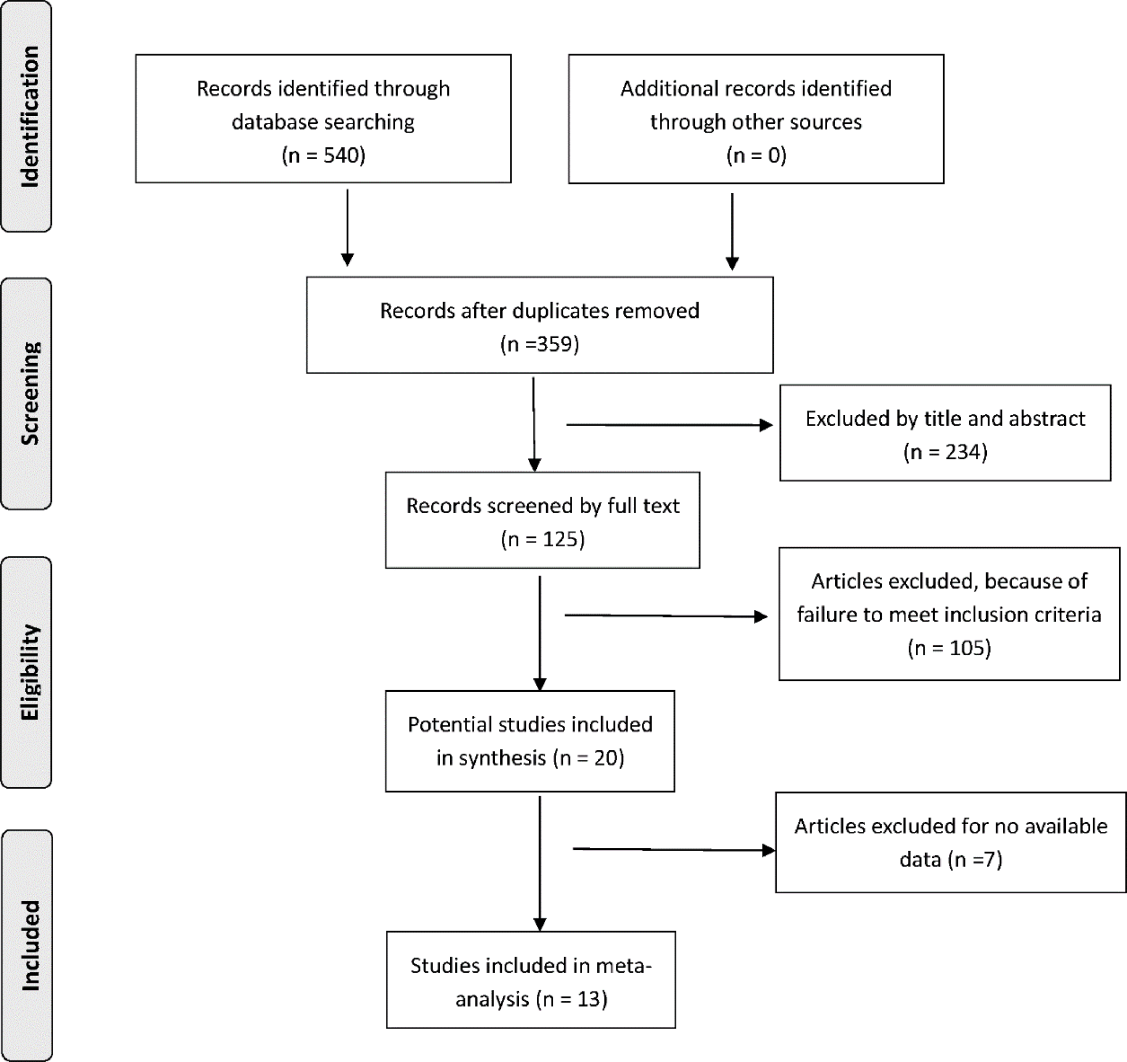
Flowchart for the identification of eligible studies.

540 relevant articles were retrieved by the approaches mentioned in the previous section. After screening by the titles and abstracts, 234 articles were excluded. The full article evaluation for the remaining 20 studies was performed, and ultimately 13 eligible articles (30–42) were included in the systematic review. The PRISMA flowchart of the study is illustrated in **Figure 1**. The principal characteristics and further details of eligible articles are shown in **Table 1**. The assessment of study quality for all included trials is summarized in **Table 2**.

**Table 1:** The principal characteristics and details of eligible articles.

Study	Gender(Male/Female)	Age (year), median (range)	Study design	Time of ^18^F-FDG PET/CT	Cancer stage	Treatment regimen	Follow-up time (month)	Endpoint
Angelo Castello 2020 (30)	23/12	77 (51-86)	Prospective	Before treatment	Advanced Metastatic	Nivolumab or pembrolizumab or nivolumab plus ipilimumab or atezolizumab	13.2 (4.9-21.6)	PFS, OS
David Chardin 2020 (31)	59/20	64 (58-72)	Prospective	Before treatment	Advanced or metastatic	Pembrolizumab or nivolumab	12.3 (6.1-19.3)	OS
Kosuke Hashimoto 2020 (32)	65/20	Not reported	Retrospective	After previous treatment and before the initiation of anti-PD-1 antibody	Advanced	Nivolumab or pembrolizumab	Not reported	PFS, OS
Romain-David Seban 2020 (33)	56/24	61.9 (34.2-84.8)	Retrospective	Baseline	IIIB/IV	Nivolumab or pembrolizumab or atezolizumab	11.6 (7.7-15.5)	PFS, OS
Romain-David Seban1 2020 (34)	38/25	65 (37-86)	Retrospective	Baseline	IV or IIIB	Pembrolizumab	13.4 (9.0-17.9)	PFS, OS
Romain-David Seban2 2020 (35)	38/25	65 (37-86)	Retrospective	Initial stage	IIIB/IV	Pembrolizumab	13.4 (9.0-17.9)	PFS, OS
Damijan Valentinuzzi 2020 (36)	15/15	65 (46-77)	Prospective	Within 4 weeks before treatment	IV	Pembrolizumab	21.4	OS
Ou Yamaguchi 2020 (37)	39/9	69 (47-86)	Retrospective	Before administration	Advanced	Pembrolizumab	11.5 (1-29.5)	PFS, OS
Angelo Castello 2021 (38)	34/16	73	Prospective	Before treatment	Advanced	Nivolumab or pembrolizumab or atezolizumab	12.4 (9.7-15.2)	PFS, OS
David Lang 2021 (39)	56/10	64 (38-81)	Retrospective	Before treatment	Advanced	Pembrolizumab plus carboplatin/pemetrexed or pembrolizumab plus carboplatin/paclitaxel	12 (10-14)	PFS, OS
Karolien Vekens 2021 (40)	17/13	67 (41.0-92.0)	Retrospective	Before start of treatment	IV	Pembrolizumab	20 (4.2-37.6)	PFS, OS
Florian Eude 2022 (41)	43/22	64.1 ± 10.5	Retrospective	Baseline	III/IV	Pembrolizumab	12	OS
Chang Gon Kim 2022 (42)	41/11	63 (33-84)	Retrospective	Before treatment	Advanced	Pembrolizumab combined with platinum-based chemotherapy	16.7 (15.7-17.7)	PFS, OS

**Table 2:** Quality assessment of included studies using NOS.

Study	Selection	Comparability	Outcome	Total points
	(1) Representativeness of exposed cohort (2) Selection of non-exposed cohort (3) Ascertainment of exposure (4) Demonstration that the outcome of interest was not present at the start of the study (☆☆☆☆)	(1) Comparability of cohorts on the basis of the design or analysis (☆☆)	(1) Assessment of outcome (2) Was follow-up long enough for outcomes to occur(3) Adequacy of follow-up of cohorts (☆☆☆)	
Angelo Castello 2020 (30)	☆1), ☆2), ☆3), ☆4)	☆☆	☆1), ☆2) , ☆3)	9
David Chardin 2020 (31)	☆1), ☆2), ☆3), ☆4)	☆☆	☆1), ☆2) , ☆3)	9
Kosuke Hashimoto 2020 (32)	☆1), ☆2), ☆3), ☆4)	☆	☆1)	6
Romain-David Seban 2020 (33)	☆1), ☆2), ☆3), ☆4)	☆	☆1), ☆2), ☆3)	8
Romain-David Seban1 2020 (34)	☆1), ☆2), ☆3) , ☆4)	☆	☆1), ☆2), ☆3)	8
Romain-David Seban2 2020 (35)	☆1), ☆2), ☆3), ☆4)	☆	☆1), ☆2), ☆3)	8
Damijan Valentinuzzi 2020 (36)	☆1), ☆2), ☆3), ☆4)	☆☆	☆1), ☆2), ☆3)	9
Ou Yamaguchi 2020 (37)	☆1), ☆2), ☆3), ☆4)	☆	☆1), ☆2)	7
Angelo Castello 2021 (38)	☆1), ☆2), ☆3), ☆4)	☆☆	☆1), ☆2)	8
David Lang 2021 (39)	☆1), ☆2), ☆3), ☆4)	☆	☆1), ☆2), ☆3)	8
Karolien Vekens 2021 (40)	☆1), ☆2), ☆3), ☆4)	☆	☆1), ☆2)	7
Florian Eude 2022 (41)	☆1), ☆2), ☆3), ☆4)	☆	☆1), ☆2)	7
Chang Gon Kim 2022 (42)	☆1), ☆2), ☆3), ☆4)	☆	☆1), ☆2), ☆3)	8

### The value of SUV_max_, SUV_mean_, MTV and TLG of ^18^F-FDG PET/CT in prediction of OS

**Figure 2:**
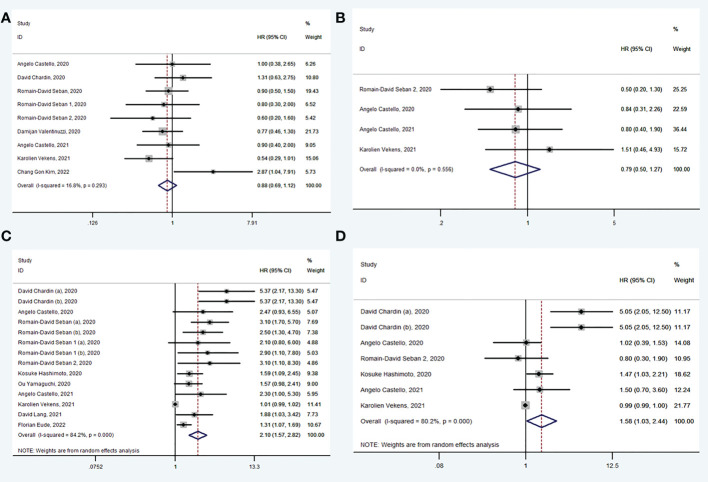
Forest plots of hazard ratios in studies investigating the prognostic value of SUVmax **(A)**, SUVmean **(B)**, MTV **(C)**, TLG **(D)** for OS.

A total of 9 studies (30, 31, 33–36, 38, 40, 42) explored the value of baseline SUV_max_ in prediction of OS. There was no significance in OS between high SUV_max_ group and low SUV_max_ group (HR: 0.88, 95% CI: 0.69-1.12) using random model for low heterogeneity between the studies (I^2^ = 16.8%, *P* = 0.293) (**Figure 2A**). Begg’s test (*P* = 0.602) manifested that there was no publication bias for recruited studies on OS.

A total of 4 studies (30, 35, 38, 40) explored the value of baseline SUV_mean_ in prediction of OS. There was no significance in OS between high SUV_mean_ group and low SUV_mean_ group (HR: 0.79, 95% CI: 0.50-1.27) using fixed model for low heterogeneity between the studies (I^2^ = 0.0%, *P* = 0.556) (**Figure 2B**). Begg’s test (*P* = 1.000) manifested that there was no publication bias for recruited studies on OS.

A total of 11 studies (30–35, 37–41) explored the value of baseline MTV in prediction of OS. In pooled analysis, OS was evidently prolonged in low MTV group (HR: 2.10, 95% CI: 1.57-2.82) using random model for heterogeneity between the studies (I^2^ = 84.2%, *P* < 0.001) (**Figure 2C**). Sensitivity analysis was performed and it was found that heterogeneity was reduced from 84.2% to 54.0% by excluding studies (Karolien Vekens, 2021), and the results were stable. Begg’s test (*P* = 1.000) manifested that there was no publication bias for recruited studies on OS.

A total of 6 studies (30–32, 35, 38, 40) explored the value of baseline TLG in prediction of OS. In pooled analysis, OS was evidently prolonged in low TLG group (HR: 1.58, 95% CI: 1.03-2.44) using random model for heterogeneity between the studies (I^2^ = 80.2%, *P* < 0.001) (**Figure 2D**). Sensitivity analysis was performed and it was found that heterogeneity was reduced from 80.2% to 69.6% by excluding studies (Karolien Vekens, 2021), and the results were stable. Begg’s test (*P* = 0.453) manifested that there was no publication bias for recruited studies on OS.

### The value of SUV_max_, SUV_mean_, MTV and TLG of ^18^F-FDG PET/CT in prediction of PFS

**Figure 3:**
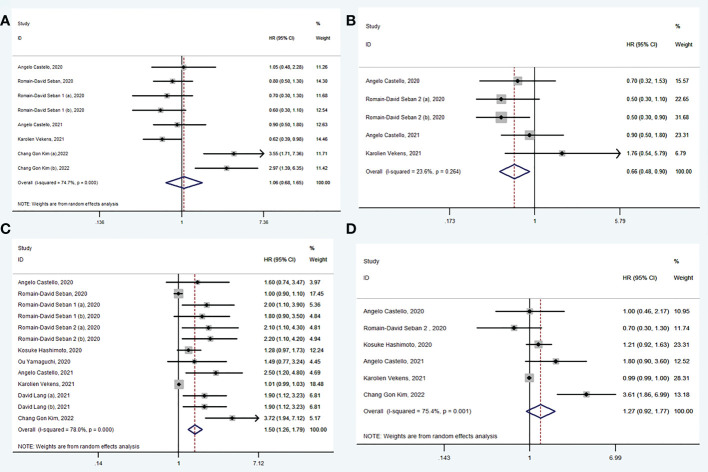
Forest plots of hazard ratios in studies investigating the prognostic value of SUVmax **(A)**, SUVmean **(B)**, MTV **(C)**, TLG **(D)** for PFS.

A total of 6 studies (33, 34, 36, 38, 40, 42) explored the value of baseline SUV_max_ in prediction of PFS. There was no significance in PFS between high SUV_max_ group and low SUV_max_ group (HR: 1.06, 95% CI: 0.68-1.65) and using random model for high heterogeneity between the studies (I^2^ = 74.7%, *P* < 0.001) (**Figure 3A**). Begg’s test (*P* = 0.174) manifested that there was no publication bias for recruited studies on FPS.

A total of 4 studies (30, 35, 38, 40) explored the value of baseline SUV_mean_ in prediction of PFS. In pooled analysis, PFS was evidently prolonged in low SUV_mean_ group (HR: 0.66, 95% CI: 0.48-0.90) using fixed model for low heterogeneity between the studies (I^2^ = 23.6%, *P* = 0.264) (**Figure 3B**). Begg’s test (*P* = 0.221) manifested that there was no publication bias for recruited studies on PFS.

A total of 10 studies (30, 32–35, 37–40, 42) explored the value of baseline MTV in prediction of PFS. In pooled analysis, PFS was evidently prolonged in low MTV group (HR: 1.50, 95% CI: 1.26-1.79) using random model for heterogeneity between the studies (I^2^ = 78.0%, *P* < 0.001) (**Figure 3C**). Sensitivity analyses showed that heterogeneity was reduced from 78.0% to 16.7% when studies were excluded (Romain-David Seban, 2020 and Karolien Vekens, 2021), and the results were stable. Begg’s test (*P* = 0.625) manifested that there was no publication bias for recruited studies on PFS.

A total of 6 studies (30, 32, 35, 38, 40, 42) explored the value of baseline TLG in prediction of PFS. There was no significance in PFS between high TLG group and low TLG group (HR: 1.27, 95% CI: 0.92-1.77) using random model for high heterogeneity between the studies (I^2^ = 75.4%, *P* = 0.001) (**Figure 3D**). Begg’s test (*P* = 0.625) manifested that there was no publication bias for recruited studies on PFS.

### Subgroup analysis

For patients with NSCLC, a subgroup analysis of the treatment line was performed in the study, which were first line (only including first line treatment) and undefined line (including first or later line treatment, and second or later line treatment). The results are shown in **Table 3**. Subgroup analysis showed that no significant differences in OS and PFS were found between high SUV_max_ group and low SUV_max_ group either in first line group (HR: 0.96, 95% CI: 0.54-1.71; HR: 1.19, 95% CI: 0.56-2.54) or in undefined line group (HR: 1.20, 95% CI: 0.81-1.77; HR: 0.87, 95% CI: 0.62-1.23). As for SUV_mean_, there was also no significant difference for OS and PFS between two group in first line group (HR: 0.82, 95% CI: 0.28-2.39; HR: 0.63, 95% CI: 0.35-1.14) and undefined line group (HR: 0.81, 95% CI: 0.44-1.50; HR: 0.81, 95% CI: 0.50-1.34). High MTV group displayed shorter OS than low MTV group in both first line group (HR: 1.97, 95% CI: 1.39-2.79) and undefined line group (HR: 2.11, 95% CI: 1.61-2.77), but high MTV group only showed an inferior PFS in first line group (HR: 1.85, 95% CI: 1.28-2.68). Low TLG group displayed a longer OS in undefined line group (HR: 1.37, 95% CI: 1.00-1.86), rather than in first line group (HR: 2.03, 95% CI: 0.78-5.26).

**Table 3:** Subgroup analysis of treatment line for OS and PFS.

Variables	Subgroup	HR (95% CI)	*P* value	Heterogeneity *P* (I^2^)
SUV_max_ for OS	Total	0.88 (0.69, 1.12)	0.283	0.293 (16.8%)
	First line	0.96 (0.54, 1.71)	0.892	0.055 (56.9%)
	Undefined line	1.20 (0.81, 1.77)	0.359	0.021 (62.4%)
SUV_mean_ for OS	Total	0.79 (0.50, 1.27)	0.335	0.556 (0.00%)
	First line	0.82 (0.28, 2.39)	0.712	0.152 (51.4%)
	Undefined line	0.81 (0.44, 1.50)	0.512	0.941 (0.00%)
MTV for OS	Total	2.10 (1.57, 2.82)	< 0.001	< 0.001 (84.2%)
	First line	1.97 (1.39, 2.79)	< 0.001	< 0.001 (84.0%)
	Undefined line	2.11 (1.61, 2.77)	< 0.001	0.428 (0.00%)
TLG for OS	Total	1.58 (1.03, 2.44)	0.038	< 0.0001 (80.2%)
	First line	2.03 (0.78, 5.26)	0.147	< 0.0001 (88.1%)
	Undefined line	1.37 (1.00, 1.86)	0.047	0.635 (0.00%)
SUV_max_ for PFS	Total	1.06 (0.68, 1.65)	0.798	< 0.0001 (74.7%)
	First line	1.19 (0.56, 2.54)	0.644	< 0.0001 (85.1%)
	Undefined line	0.87 (0.62, 1.23)	0.437	0.838 (0.00%)
SUV_mean_ for PFS	Total	0.67 (0.47, 0.97)	0.033	0.264 (23.6%)
	First line	0.63 (0.35, 1.14)	0.565	0.146 (48.0%)
	Undefined line	0.81 (0.50, 1.34)	0.401	0.628 (0.00%)
MTV for PFS	Total	1.50 (1.26, 1.79)	< 0.001	< 0.0001 (78.0%)
	First line	1.85 (1.28, 2.68)	0.001	< 0.0001 (81.9%)
	Undefined line	1.31 (0.95, 1.81)	0.102	0.020 (69.6%)
TLG for PFS	Total	1.27 (0.92, 1.77)	0.148	0.001 (75.4%)
	First line	1.60 (0.32, 8.00)	0.125	0.001 (90.6%)
	Undefined line	1.08 (0.90, 1.30)	0.417	0.191 (36.8%)

First line: only including first line treatment; undefined line: including first or later line treatment, and second or later line treatment.

## Discussion

PET/CT has been proved to be of great value in the diagnosis, clinical staging, radiotherapy localization, efficacy evaluation and prognosis of NSCLC (31, 43), but its prognostic status in ICIs treatment is unclear. This meta-analysis evaluated the prognostic value of baseline metabolic parameters of ^18^F-FDG PET/CT for advanced or metastasis NSCLC by analyzing the HR of PFS and OS in patients with high SUV_max_, SUV_mean_, MTV or TLG versus those with low SUV_max_, SUV_mean_, MTV or TLG. The pooled results showed patients with high MTV and TLG prior to ICIs treatment may had shorter OS than patients with lower metabolic rate, suggesting that MTV and TLG may be meaningful prognostic markers. High MTV may also predict worse PFS, and patients with high SUV_mean_ had better PFS. No correlation was found between SUV_max_ and OS or PFS.

Unlike traditional tumor therapy by killing cancer cells, ICIs work in an immunomodulatory way maybe by modulating tumor metabolism. The proliferation rate of tumor cells is positively related to their glucose uptake (44). Higher uptake of FDG leads to more aggressive tumors and worse prognosis (45). SUV has independent and prognostic value for OS in NSCLC patients, but most studies are based on early NSCLC (46–48). SUV_max_ and SUV_mean_ are associated with the presence of CD8^+^ TILs in patients with NSCLC (20). Takada et al. also linked high SUV_max_ to positivity for PD-L1 (49). These findings suggest that high FDG uptake may indicate an immunologically cold tumor. However, previous studies have also found that this may not be true for patients with advanced NSCLC. SUV_max_ is, to a lesser extent, considered to be an effective predictor of prognosis in patients with NSCLC, regardless of treatment or disease stage (50, 51). Our pooled results also found that baseline SUV_max_ did not provide any prognostic information. High SUV_mean_ may predict longer PFS, but had no correlation with OS. The results of subgroup analysis showed no predictive value of SUV_max_ and SUV_mean_ in first line group and undefined line group. It is worth noting that pseudoprogression can lead to possible misjudgment by PFS. Both SUV_max_ and SUV_mean_ can represent the uptake level of FDG, but cannot reflect the overall metabolism of tumor. Especially, SUV does not consider the effect of tumor volume on prognosis, and only reflects the metabolic level of mononectin in the lesions (52, 53).

When extensive metastasis occurs, the tumor load is large, which may be more valuable than the metabolic activity for primary lung cancer prognosis. Liao S et al. (54) found that MTV and TLG were better predictors of clinical outcomes in patients with stage IV NSCLC than SUV_max_ and SUV_mean_. MTV and TLG are another type of metabolic parameters that take tumor metabolic volume and metabolic activity into comprehensive consideration. MTV can not only calculate the tumor volume, but also display the metabolic activity of the lesions. However, TLG combined with MTV and glycolysis can analyze the overall metabolic status of lesions more comprehensively. The pooled results of this study showed OS and PFS was shortened in high MTV group, indicating the larger the tumor metabolic volume and metabolic load, the shorter the possible survival, the worse the prognosis. High TLG group also showed shorter OS than low TLG group, but no significance was found for PFS. Nonetheless, significant heterogeneity was found in the analysis of MTV and TLG predicting PFS and OS. The subgroup analysis of treatment line showed that OS in high MTV group was significantly shorter than that in low MTV group in either first line group or undefined line group. However, high MTV may only predict poor PFS in first line group, but not in undefined line group. Meanwhile, high TLG was associated with poor OS in undefined line group, but not in first line group. MTV has shown good prognostic ability, but the predictive significance of TLG is unclear in the different treatment lines.

There is no theoretical explanation of the mechanism by which these metabolic parameters serve as prognostic markers for ICIs treatment. Advanced or metastatic NSCLC is often accompanied by lymph node metastasis, or liver, bone, brain and other organ metastasis. Therefore, the impact of metastasis other than the primary site of tumor should be focused on in prognosis assessment. Systemic tumor metabolic load includes the metabolic load of primary tumor, metastatic lymph node and distant metastasis, which more comprehensively reflects systemic metabolic information. MTV and TLG measure tumor volume and metabolic activity in three dimensions, providing more information about tumor aggressiveness. This study shows that NSCLC patients with high baseline MTV have a poor prognosis after ICIs treatment. MTV reflects the real volume of tumors with high metabolic activity in tumor tissues, and the high accumulation of FDG may be related to tumor necrosis or hypoxia caused by larger tumor deposition. Tumor necrosis, acidity and hypoxia can promote the establishment of immunosuppressive tumor microenvironment by recruiting a variety of immunosuppressive cells, such as myeloid-derived suppressor cells, regulatory t cells (Tregs) and tumor-associated macrophages (55, 56). These are closely associated with tumor recurrence and survival deterioration (57, 58). In this environment, FOXP3^+^, the major regulator of Tregs, has been reported to be positively correlated with MTV (59, 60). Whereas, in the anoxic environment caused by tumor necrosis and inflammation, no correlation has been found between PD-L1 expression and MTV (61). Studies have found that NSCLC patients with positive PD-L1 expression have significantly higher MTV than patients with negative PD-L1 expression (62), While other studies have shown that MTV is not significantly associated with PD-L1 expression (60, 63). Although clinical trials have confirmed that high expression of PD-L1 can make tumor cells more sensitive to immunotherapy (64, 65), and can also mediate immune escape (66, 67). PD-L1 may play a dual role, but it is vague what mechanism determines PD-L1 to activate drug resistance or response pathways. In summary, an environment with high MTV may form an immunosuppressive state, thus leading to drug resistance of ICIs, regardless of PD-L1 expression. MTV may be a better parameter to predict immunotherapy response and prognosis, but its mechanism needs to be clarified.

This is the first meta-analysis to evaluate ability of baseline metabolic parameters of ^18^F-FDG PET/CT for survival in advanced or metastatic NSCLC patients treated with ICIs. Our results suggest that MTV can be considered as a basis for patient stratification in the immunotherapy and prognosis of advanced or metastatic NSCLC patients. This provides some data support for clinical application of ^18^F-FDG PET/CT to judge the prognosis of NSCLC patients and guides the treatment of lung cancer. Although analysis software is available to measure these metabolism parameters (40–42), MTVand TLG are not typical components of standard PET/CT reports because of insufficient evidence of clinical application value and increased workload. As the clinical significance of these parameters is fully validated, radiologists will become aware of their potential applications. It is economical and feasible to help patients select the best treatment strategy by obtaining baseline MTV and TLG of NSCLC patients only once before ICIs treatment in the future. Early identification of patients who do not benefit from immunotherapy may lead to timely decisions and discontinuation of treatment, potentially reducing drug toxicity and the high cost of immunotherapy and initiating different treatment regimens earlier.

However, the study has several limitations. Firstly, heterogeneity was detected in the present meta-analysis. This may be due to bias in detection methods, differences in ICIs treatment, etc. Secondly, we cannot determine an optimal threshold to classify volume parameters as high or low. Different cut-off values and delineation strategies, as well as different histological methods, may influence the occurrence and survival of events. Thirdly, there are different types of NSCLC, such as adenocarcinoma and squamous cell carcinoma, with different prognosis. However, in the currently included studies, all of these subtypes are mixed and these studies can yield different results in response prediction and monitoring with ^18^F-FDG PET/CT imaging. Therefore, further large-scale prospective studies are necessary to evaluate whether MTV and TLG can be independent prognostic factors for clinical outcome in patients with different types of NSCLC.

## Conclusion

Baseline MTV may have strong predictive power for survival, while baseline SUV_max_ and SUV_mean_ may not be appropriate prognostic markers in advanced or metastatic NSCLC patients treated with ICIs. Baseline TLG may predict OS for only a subset of patients, and its predictive value remains to be examined.

## Data availability statement

The raw data supporting the conclusions of this article will be made available by the authors, without undue reservation.

## Author contributions

TL and LZ conducted the literatures screening. TL and RP conducted the statistical analysis. TL, LZ and LH wrote the manuscript. LH and CY revised the manuscript. All authors contributed to the article and approved the submitted version.

## Conflict of interest

The authors declare that the research was conducted in the absence of any commercial or financial relationships that could be construed as a potential conflict of interest.

## Publisher’s note

All claims expressed in this article are solely those of the authors and do not necessarily represent those of their affiliated organizations, or those of the publisher, the editors and the reviewers. Any product that may be evaluated in this article, or claim that may be made by its manufacturer, is not guaranteed or endorsed by the publisher.
